# Insights into uranium enrichment of indigenous electroactive *Shewanella putrefaciens*

**DOI:** 10.3389/fmicb.2025.1731432

**Published:** 2026-01-29

**Authors:** Guolin Yang, Ling Wei, Liang Liu, Bo Mu, Tao Chen

**Affiliations:** 1 Institute of Basic Medicine and Forensic Medicine, Nuclear Medicine and Radiation Safety Key Laboratory of Sichuan Province, North Sichuan Medical College, Nanchong, Sichuan, China; 2 Department of Agricultural Science and Technology, Nanchong Vocational and Technical College, Nanchong, Sichuan, China; 3 CAEA Innovation Center of Nuclear Environmental Safety Technology, School of National Defense and Nuclear Science and Technology, Southwest University of Science and Technology, Mianyang, Sichuan, China

**Keywords:** bioremediation, isolation, microbial diversity, *Shewanella putrefaciens*, uranium

## Abstract

Bioremediation of uranium-contaminated environments using native bacteria shows great promise. While *Shewanella putrefaciens* (*S. putrefaciens*) is a known uranium reducing bacterium, the mechanisms and adaptability of indigenous strains from uranium mine tailings remain unexplored. This study isolated a dominant indigenous strain of *S. putrefaciens* and employed a combined genomic and spectroscopic approach to elucidate its unique uranium fixation mechanism. Microbial diversity analysis confirmed the dominance of *Shewanella* in the oligotrophic and radioactive tailings. Whole-genome sequencing revealed a significant enrichment of genes related to energy metabolism and stress resistance, providing a genetic basis for its survival and activity. Crucially, by combining advanced spectroscopic techniques with an MtrA gene knockout experiment, we deciphered the specific role of the Mtr pathway in extracellular electron transfer for uranium reduction. Remarkably, the isolated strain achieved a uranium removal efficiency of up to 93% under experimental conditions, demonstrating its high potential for uranium bioremediation. This work not only provides a robust indigenous candidate for bioremediation but also delivers novel mechanistic insights into the uranium transformation processes of indigenous *Shewanella*, advancing strategies for the application of tailored microbiomes in radioactive waste management.

## Graphical Abstract

**Figure:**
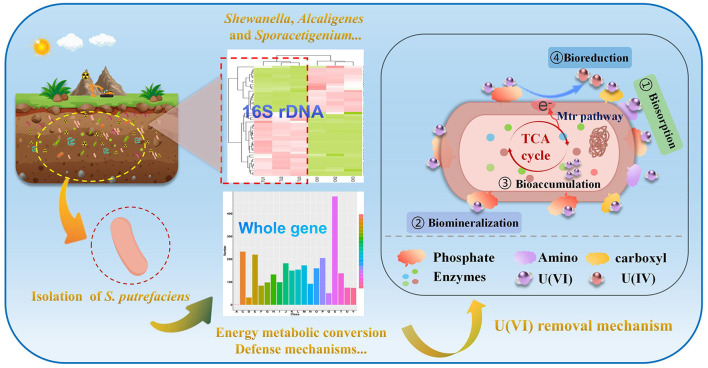

## 1 Introduction

Uranium is a naturally occurring radioactive metal that plays a vital role in nuclear energy, radiological medicine, irradiation preservation, and mutagenic breeding (Chen et al., 2021; Lin et al., 2024). However, processes such as uranium mining and smelting, uranium enrichment and component manufacturing may release a certain amount of uranium into the environment, posing risks to ecosystems and human health (Lv et al., 2023; Richardson et al., 2022; Wang et al., 2022). In the face of the increasingly serious situation of soil uranium contamination, the development of efficient uranium contaminated soil remediation technology has become an urgent task (Zhang Y. et al., 2023). Compared with traditional remediation methods, bioremediation technology is considered to be a promising remediation technology for radioactively contaminated soil due to its easy operation, low cost and no secondary pollution, especially for the remediation of surface soil with a large contaminated area and a light degree of contamination (Li R. et al., 2024). Microbial bio-enrichment is considered an alternative technology for *in situ* remediation of uranium-contaminated environments due to the immobilization capacity of microorganisms for heavy metals (Chen et al., 2024).

Microorganisms are ubiquitous in environment and characterized by high reproductive rates, making them sensitive bioindicators that promptly reflect soil or water ecological health (Bach et al., 2018; Handelsman and Wackett, 2002). The abundance and diversity of microbial communities may serve as indicators for assessing ecosystem health, thereby offering valuable insights for ecosystem restoration (Jiang et al., 2020; Pajares and Bohannan, 2016). Moreover, prolonged environmental domestication has enabled microorganisms to evolve adaptive physiological metabolism, thereby mitigating external stressors (Domeignoz-Horta et al., 2023; Zhou et al., 2023). These adaptive traits underscore their significant role in ecological remediation and regulation, particularly in the context of heavy metal pollution (Xi et al., 2024; Xuan et al., 2024). Microorganisms can repair heavy metal-contaminated water or soil through multiple pathways, such as bioreduction, bioaccumulation, biosorption, and biomineralisation, which collectively reduce the bioavailability and toxicity of metals (Banala et al., 2021; Verma and Kuila, 2019; You et al., 2021). In recent years, researchers have isolated a variety of uranium-tolerant bacteria from uranium-contaminated soils or waters, including *Thiobacillus, Bacillus, Arthrobacter, Enterobacter, Geobacter, Shewanella*, etc. (Li Q. et al., 2024; Li et al., 2017; Lu et al., 2024). Uranium mining activities create unique habitats characterized by multiple environmental stresses, such as low pH, heavy metal toxicity, and radionuclide toxicity, which significantly influence the structure of local microbial communities (Liu et al., 2024). Notably, in such extreme environments, microbial groups possessing metal reduction capabilities (e.g., the genus *Shewanella*) often become dominant populations, with their exceptional metabolic flexibility considered a key adaptive strategy (Hau and Gralnick, 2007; Lashani et al., 2023). However, research on the genomes and uranium removal mechanisms of indigenous functional strains currently screened from uranium tailings remains insufficient. Therefore, isolating and screening native bacteria with highly efficient uranium adsorption and tolerance, and analyzing their molecular mechanisms of uranium enrichment, are of great significance for the *in-situ* treatment of uranium contaminated soil or water and soil.

Herein, we systematically analyzed the microbial diversity in soils from uranium and non-uranium mining areas by high-throughput sequencing technology. The diversity characteristics of the microbial community in uranium mining soils were systematically revealed, and a uranium-tolerant dominant strain (*Shewanella putrefaciens, S. putrefaciens*) was successfully isolated and identified. In addition, the whole genome of *S. putrefaciens* was analyzed, and its morphology and compositional structure before and after uranium enrichment were characterized, which in turn revealed the potential mechanism of its uranium enrichment. These results not only help to deepen the understanding of microecological effects in uranium mining areas, but also provide an important reference for the *in situ* ecological remediation of uranium-contaminated areas.

## 2 Materials and methods

### 2.1 Reagents and materials

Uranyl nitrate hexahydrate (UO_2_(NO_3_)_2_·6H_2_O) was purchased from Hubei Chushengwei Chemistry Co., Ltd. Other standard reagents were all acquired from Aladdin Industrial Corporation. LB medium contained 10 g/L tryptone, 5 g/L of yeast extract, and 10 g/L of NaCl, adjust the pH to 7.0 with 5 mol/L NaOH and steam sterilize at 121 °C for 20 min.

### 2.2 Sample collection and 16S rRNA sequencing

Topsoil samples were collected from a uranium tailings pond (designated as TU) located in Southwest China and a nearby non-uranium mining area (designated as CK) for comparison. Three biological replicates were collected for each site. The samples were screened through a 2 mm sieve to remove impurities such as grass roots and gravel, and then partitioned. For microbial community analysis, a soil subsample was stored at −80 °C until DNA extraction. For the isolation of viable bacteria, a separate, freshly collected soil aliquot was processed within 24 h without being subjected to freezing. The frozen samples were subsequently preserved in dry ice and dispatched to OE Biotech Co., Ltd. (Shanghai, China) for 16S rRNA sequencing analysis. Statistical significance was evaluated using Wilcoxon rank-sum test, with *p* < 0.01 considered significant.

### 2.3 Isolation and purification of uranium-resistant strains

For bacterial isolation, 10 g of the fresh TU sample was placed in 90 mL of sterile water, shaken well for 30 min, then diluted in gradients of multiples of 10^−5^ and the diluted suspension was spread on LB containing 100 mg/L uranium selective plates. After 48 h of incubation at 37 °C and at least three purifications were performed on LB plates using the scratch method. Subsequently, the isolated bacteria were inoculated into LB liquid medium at 30 °C for 24 h. The activated bacteria were separated by centrifugation and then inoculated into filtered and sterilized LB liquid medium [containing 100 mg/L U(VI)] and incubated at 30 °C and 150 rpm, and the absorbance value of the culture solution was measured at 600 nm with Microbial Growth Curve Monitor. Unless otherwise specified, the pH of the LB medium used in the experiment is maintained at 7.0 (adjusted using 0.01 M NaOH or HCl).

### 2.4 DNA extraction, sequencing, and analysis

The detailed protocols for DNA extraction, 16S rRNA gene amplification, sequencing, and bioinformatic analysis are provided in Supplementary Section S1. The complete 16S rRNA gene sequence of A-1 (*S. putrefaciens*) to the NCBI GenBank database (ID: PX705773).

### 2.5 Whole genome analysis of *S. putrefaciens*

Whole genome sequencing was performed on genomic DNA extracted from a pure, overnight culture of the isolated *Shewanella putrefaciens* strain A-1. The bacterial cells were harvested during the late-exponential growth phase. Whole genome sequencing was performed by OE Biotech Co., Ltd. using second-generation sequencing technology, combined with Illumina NovaSeq and Oxford Nanopore ONT sequencing platforms to sequence the constructed gene libraries. The sequencing data were subjected to strict quality control, including removal of splice sequences, low-quality reads, short reads, and filtering treatment of fuzzy base N. The sequencing data were then processed using the UniCycler (Wick et al., 2017). Subsequently, genome assembly was performed on the filtered data using UniCycler and Flye software (Kolmogorov et al., 2019; Wick et al., 2017). To ensure the accuracy of the assembly, Pilon software was further used to correct the assembly results (Walker et al., 2014), and the complete genome sequence was finally obtained. In addition, the whole genome information was analyzed for functional annotation and prediction based on KEGG (http://www.genome.jp/kegg) and COG (http://www.ncbi.nlm.nih.gov/COG) databases.

### 2.6 Uranium removal experiment by *S. putrefaciens*

*S. putrefaciens* was cultured after 24 h, centrifuged at 8,000 rpm for 10 min to collect the bacterial precipitate, and then washed three times with 0.9% NaCl solution to avoid the influence of the medium components for the uranium removal experiment. A uranium stock solution (1.0 g/L) was prepared by dissolving uranyl nitrate hexahydrate (UO_2_(NO_3_)_2_·6H_2_O) in deionized water and filter-sterilized (0.22 μm). Specifically, 1 ml of this solution was added to a serum vial containing 9 mL of cell suspension (containing 0.9% NaCl). Subsequently, the suspension was sealed and reacted at 30 °C with continuous stirring (150 rpm), and the solution was aspirated at different time intervals and then filtered through a 0.2 μm microporous filter membrane to remove the bacteria. Arsenazo III was utilized as a dye and the concentration of $\text{U}O_{2}^{2+}$ was measured by UV-vis absorption spectroscopy at a wavelength of 651.8 nm (Bhatti et al., 1991). The removal efficiency (*R*) and adsorption capacity (*Q*) of uranium were calculated using the following equations: *R*(%) = (*C*_0_ – *C*)/*C*_0_ × 100% and *Q*(mg/g) = (*C*_0_ – *C*) × V/m. where *C*_0_ (mg/mL) and *C* (mg/mL) represent the initial and real-time concentrations of U(VI), respectively, V(L) is the volume of the reaction solution, and m(g) is the dry mass of the bacterial cells (DCW). Throughout this study, bacterial concentrations are expressed in terms of g/L unless otherwise stated. A calibration curve between OD_600_ and DCW was established for accurate conversion. All experiments were performed in triplicate.

### 2.7 Characterization

Characterization of bacterial samples after uranium loading (including SEM, TEM-EDS, FT-IR, XRD, and XPS) was performed using biomass collected after 12 h of reaction in a 0.9% NaCl solution containing 100 mg/L U(VI). Complete experimental procedures and instrument parameters are detailed in Supplementary Section S2.

### 2.8 The *S. putrefaciens*-ΔMtrA mutants deficient construction

The MtrA knockout mutant (ΔMtrA) was generated via homologous recombination using the suicide vector pDS3.0. Briefly, approximately 550 bp of upstream and downstream flanking regions of the MtrA gene were amplified from *S. putrefaciens* genomic DNA. These fragments were ligated into SacI-linearized pDS3.0 and transformed into *E. coli* WM3064. The resulting recombinant plasmid was introduced into wild-type *S. putrefaciens* by conjugation. Merodiploids were selected on VBMM agar containing gentamicin (10 μg/mL), followed by counter-selection on VBMM agar with 15% sucrose to obtain double-crossover mutants. Gene deletion was confirmed by PCR and sequencing of the target locus. The mutant strain (ΔMtrA) was subsequently used in uranium removal experiments to evaluate the role of the Mtr pathway in extracellular electron transfer.

## 3 Results and discussion

### 3.1 Characterization of soil bacterial communities in uranium mines

**Figure 1:**
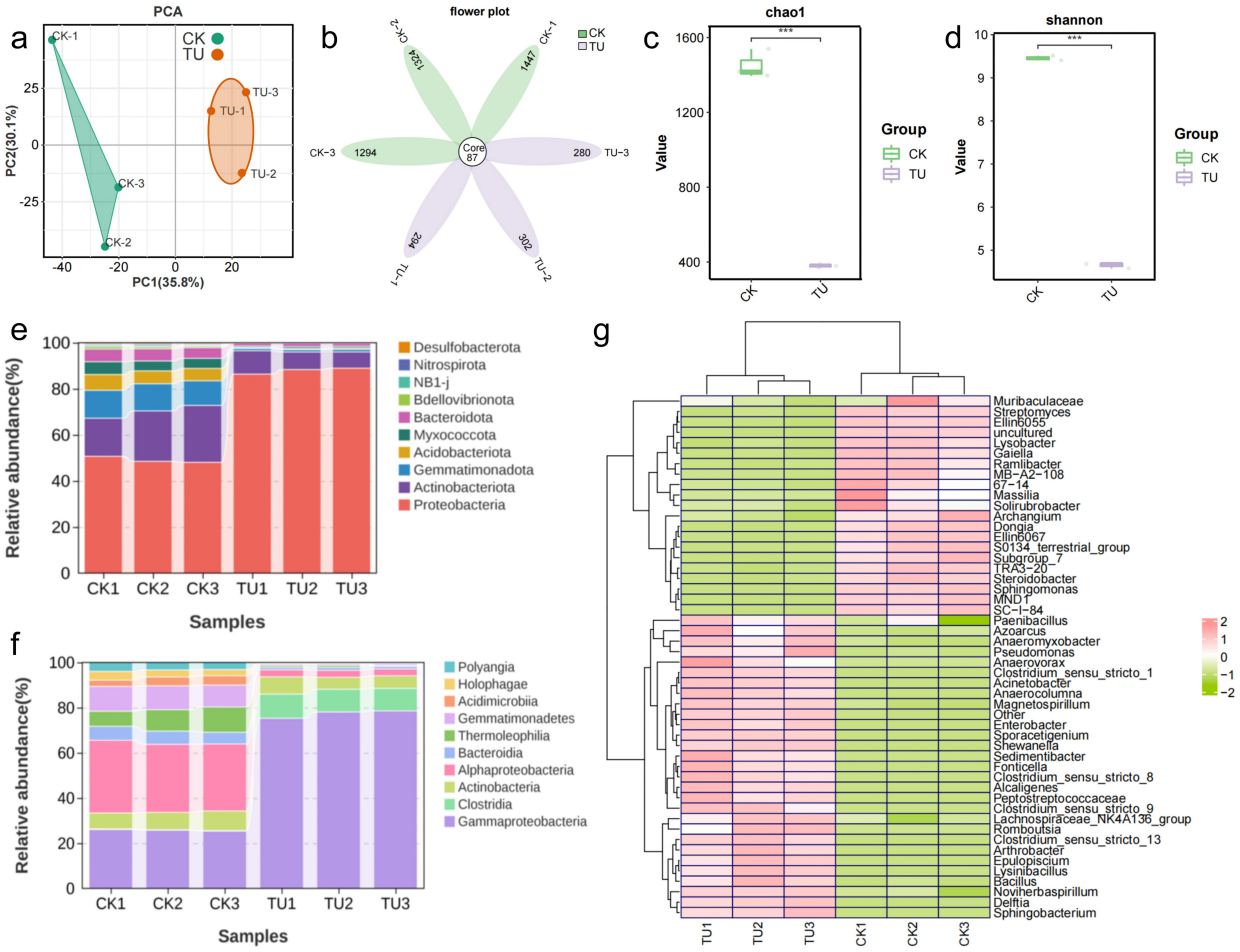
Bacterial community analyses of CK and TU. **(a)** PCA analyses; **(b)** Venn diagram analyses; **(c)** Chao1 index analyses; **(d)** Shannon index analyses; **(e)** the top 10 phylum level; **(f)** Top 10 class level; **(g)** top 50 genus level heatmaps.

To investigate the structural characteristics of bacterial communities in uranium and non-uranium soils, 16S rDNA sequencing was performed. Due to the high sequencing depth (average 80,342 reads per sample), quality control of the raw data was performed. After quality control filtering, 75,716 valid data were obtained, with a quality control effectiveness rate of 94.21%. The valid sequences were analyzed by amplicon sequence variants (ASV) clustering, and the results are shown in Supplementary Figure S1. With the gradual increase of sequencing depth, the number of ASVs showed a significant growth trend. When the curve gradually leveled off and the number of ASVs no longer increased significantly, this indicated that the collected sample size was sufficiently adequate, thus verifying the reasonableness and reliability of the sequencing data (Nearing et al., 2022). In addition, Supplementary Figure S2 reflects the sequencing coverage, with both CK (non-uranium mining soil) and TU (uranium mine topsoil) above 0.99, indicating that the sequencing data are sufficient to reflect sample variation. According to the results of principal component analysis (PCA), CK and TU were clustered into two differentiated taxa, indicating significant differences in bacterial communities between CK and TU (Figure 1a). In addition, the beta-diversity analysis showed that CK and TU were better distinguished whether based on unweighted-unifrac (unweighted-unifrac) or weighted-unifrac (weighted-unifrac) distance analysis, in which bluer color indicates closer distance and higher similarity between samples, and redder color indicates farther distance (Supplementary Figures S3, S4). In order to explore the similarity between the components of CK and TU soil samples, the Venn diagram between the samples was plotted, as shown in Figure 1b. There are the same 87 species in CK and TU soil samples, and the microbial population in the CK group is significantly higher than that of the TU group. This may be due to the relatively nutrient-poor soil of the uranium mining area, which is not conducive to microorganisms' growth (Chen et al., 2025). The alpha diversity of the bacterial community was assessed by analyzing the Chao1 index and Shannon index. The former reflects the richness of the bacterial community and the latter reflects the diversity of the bacterial community. According to Figures 1c, d, Chao1 index was significantly different between the two groups, with CK had higher abundance of bacterial community than TU. Similarly, the variation in Shannon's index supports this result. In summary, there are significant differences in soil microbial communities between CK and TU, with the CK group having a significantly higher number of microorganisms than the TU group, consistent with previous reports on microbial communities in other uranium-bearing soils (Bondici et al., 2013; Wang et al., 2025). This may be attributed to the nutrient-poor conditions and metal stress [such as U, thorium (Th), cadmium (Cd) and lead (Pb)] in uranium-contaminated soils, which suppress microbial biomass and diversity (Qu et al., 2022; Wang et al., 2024; Xiang et al., 2020).

In order to better study the changes in the community distribution of microorganisms in uranium topsoil and non-uranium soil, the heatmaps of the top 10 phylum and phylum taxonomic overlays as well as the top 50 genus levels were analyzed, respectively. As shown in Figure 1e, the top 5 at the phylum level were *Proteobacteria, Actinobacteriota, Gemmatimonadota, Firmicutes*, and *Acidobacteriota*, each with an Abundance 47.1% to 79.8%, 6.3% to 24.1%, 1.0% to 11.8% and 3% to 11.7%, respectively. The above results indicate that *Proteobacteria* and *Actinobacteria* are the dominant phyla in TU and CK, which are widely present in other soil environments according to previous reports (Dhal et al., 2011; Li N. et al., 2024). As shown in Figure 1f, the top 5 are *Gammaproteobacteria, Alphaproteobacteria, Actinobacteria, Clostridia*, and *Gemmatimonadetes* in the sequential classification. Among them, *Gammaproteobacteria* has the highest abundance in the TU with the highest abundance of more than 70%. In addition, to further analyze the differences in microbial community composition between the two soils, the top 50 genera of bacterial species were clustered using heatmaps (Figure 1g). The results demonstrated that *Shewanella, Alcaligenes, Sporacetigenium, Arthrobacter* and *Acinetobacter* were the dominant genera in the uranium soil. Furthermore, differential species were annotated using Linear discriminant analysis of effect sizes (LEfSe). As shown in Supplementary Figure S5, 61 significantly different bacterial species were out in both environments based on the LDA threshold of 4.0 (*p* < 0.05). Among them, the top 3 species with high relative abundance of TU at the family level were *Shewanellaceae, Entero bacteriaceae and Alcaligenaceae*, while the top 3 in CK were *Sphingomonadaceae, Nitrosomonadaceae and Gemmatimonadaceae*. In conclusion, the above analysis indicates that there are significant differences in microbial diversity of different soil environments. This difference mainly stems from the complexity and diversity of soil environments, which leads to different distribution characteristics of microbial species in different soil types (Tong et al., 2023; Zhang Z. et al., 2023). In view of these, the screening of indigenous strains with uranium tolerance plays an important role in the remediation of uranium-contaminated environments.

### 3.2 Isolation and identification of *S. putrefaciens*

**Figure 2:**
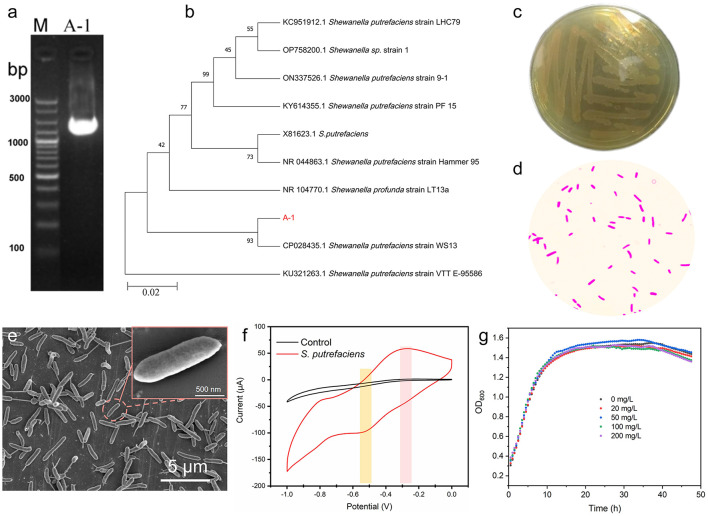
Identification of *S. putrefaciens*. **(a)** 16S rRNA gene amplification electrophoresis map of A-1; **(b)** evolutionary development tree of A-1; **(c)** colony morphology of *S. putrefaciens*; **(d)** Gram-stained microscopy of *S. putrefaciens*; **(e)** SEM of *S. putrefaciens* (inset: SEM magnification of a single bacteria); **(f)** CV curve of *S. putrefaciens*; **(g)** growth curves of *S. putrefaciens* at different uranium concentrations.

To further screen the indigenous microorganisms of uranium mining soil, a uranium-tolerant dominant bacterial strain (named A-1) was isolated from uranium mining soil by isolation and purification. And the 16S rDNA identification technique was used to identify the species of the screened dominant bacteria. As shown in Figure 2a, a DNA fragment of approximately 1.5k bp in length was successfully obtained by PCR amplification of 16S rDNA using specific universal primers. The PCR products were further sequenced and analyzed, and the length of the sequence was 1,494 bp (Supplementary Figure S6). The sequences were match analyzed in the ribosomal database (https://blast.ncbi.nlm.nih.gov/) and A-1 was identified as *Shewanella putrefaciens* (*S. putrefaciens*). Furthermore, phylogenetic analysis based on the 16S rRNA gene is primarily used for genus-level identification of strains, a widely adopted taxonomic method in the characterization of environmental isolates. Therefore, we constructed a phylogenetic tree using closely related model strains of the genus *Shewanella* to clarify the taxonomic position of strain A-1 within this genus. As shown in Figure 2b, the phylogenetic tree generated using MEGA11 software revealed that strain A-1 shared over 99% sequence similarity with the *Shewanella putrefaciens* WS13 (CP028435.1). These results strongly confirm that the isolated indigenous uranium-resistant strain A-1 belongs to *S. putrefaciens*.

The morphological characteristics of the isolated *S. putrefaciens* were examined on LB agar plates. As shown in Figure 2c, the colonies of *S. putrefaciens* exhibited a smooth surface and neat edges, appearing as opaque light pink. After further Gram staining (Figure 2d), the microscopic bacterial cells were rod-shaped and red, confirming the Gram-negative nature of the isolated *S. putrefaciens* (Müller et al., 2022). Furthermore, Scanning electron microscopy (SEM) clearly observed that *S. putrefaciens* was in the form of long rods, about 2–4 μm, with uniform and full individuals (Figure 2e). Meanwhile, the bacteria grew independently of each other without adhesion. The growth curve of *S. putrefaciens* is shown in Supplementary Figure S7. The strain entered the exponential phase at around 12 h and reached the stationary phase at 24 h. The cyclic voltammetry (CV) technique can be used to determine the electron transfer capacity of microorganisms by monitoring changes in the potential of the active substance in the reaction system (Turick et al., 2019). Therefore, electrochemical activity of *S. putrefaciens* was evaluated using the CV technique. Interestingly, as shown in Figure 2f, *S. putrefaciens* showed oxidation and reduction peaks at −0.3 V and −0.45 V, respectively, indicating that *S. putrefaciens* has a redox capacity, similar to previous reports (Okamoto et al., 2013; Yang et al., 2020). In addition, to further investigate the tolerance of *S. putrefaciens* to uranium, the growth of the bacteria was observed at uranium concentrations of 0–200 mg/L. As shown in Figure 2g, the growth activity of the strain was not affected when incubated with uranium concentration up to 200 mg/L, confirming that *S. putrefaciens* has good tolerance to uranium.

### 3.3 Uranium removal of *S. putrefaciens*

**Figure 3:**
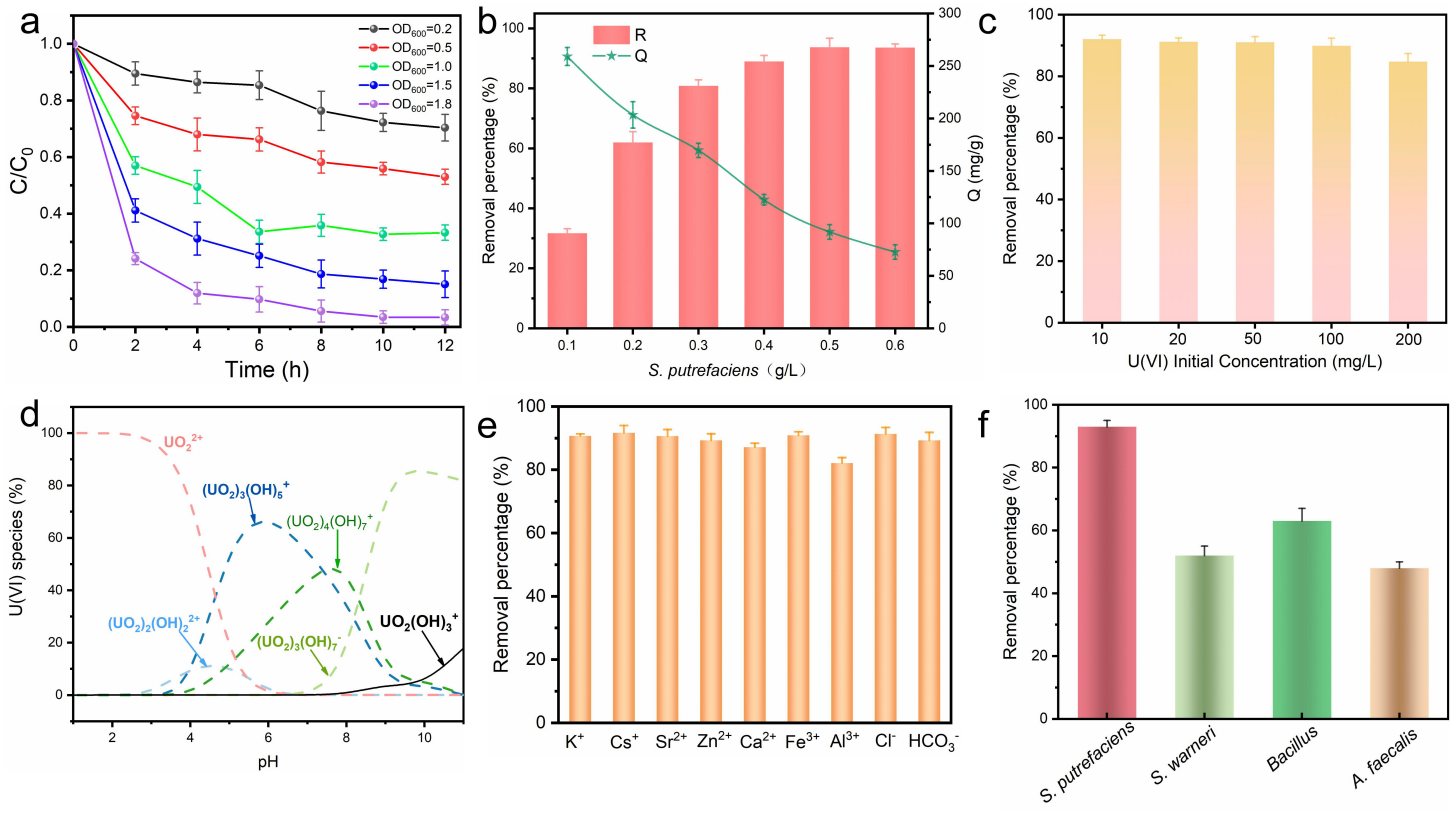
Uranium removal performance of *S. putrefaciens*. **(a)** Effect of different OD_600_; **(b)** effect of *S. putrefaciens* concentration (R: The removal efficiency of uranium; Q: The adsorption capacity of uranium); **(c)** effect of uranium initial concentrations; **(d)** the distribution of uranium species vs. pH calculated; **(e)** effect of different interfering ions; **(f)** comparison of uranium removal efficiency among four indigenous uranium-tolerant bacterial strains isolated from uranium mine soil.

To further evaluate the capacity of *S. putrefaciens* to remove uranium, batch experiments for uranium removal were conducted with different concentrations (OD_600_ value) of bacteria. As shown in Figure 3a, the uranium removal increased from 30% to 95% when the OD_600_ value of the bacterial solution increased from 0.2 to 1.8 with the increase of bacterial concentration, indicating that the increase of bacterial concentration significantly enhanced the uranium removal ability. In addition, the kinetics of uranium removal was highest in the first 2 h, which may be closely related to the adsorption mechanism of microorganisms. The functional groups on the microbial surface (e.g., carboxyl groups, phosphate groups, etc.) were able to rapidly combine with uranium to form stable complexes, thus achieving rapid removal. However, with the passage of time, the adsorption sites on the microbial surface gradually reach saturation, leading to a gradual decrease in the uranium removal rate (Guo et al., 2025). The effect of *S. putrefaciens* concentration on uranium removal efficiency was further investigated (Figure 3b). In the range of *S. putrefaciens* concentration from 0.1 to 0.5 g/L (DCW), the removal of uranium increased significantly with the increase of bacterial concentration, and the adsorption capacity decreased with increasing bacteria concentration. Although the increase in bacterial concentration provides more adsorption sites, the initial concentration of uranium (100 mg/L) remains constant. Therefore, when the adsorption sites of bacteria reach saturation, the removal efficiency of bacteria per unit mass decreases accordingly. For example, at 0.1 g/L and 0.6 g/L, the adsorption capacity of uranium is 259 mg/g and 72 mg/g, respectively. In addition, the removal performance of *S. putrefaciens* was systematically evaluated under different uranium initial concentration conditions. As shown in Figure 3c, when the initial concentration of uranium was between 10 and 100 mg/L, the removal rate of uranium by *S. putrefaciens* could be stably maintained at more than 90%. And even when the initial concentration was as high as 200 mg/L, the removal efficiency of *S. putrefaciens* can still be as high as 84.7%. This result indicates that *S. putrefaciens* has a good removal capability over a wide range of uranium concentrations.

Furthermore, the complex and diverse nature of uranium mining sites necessitated analysis of the factors influencing uranium removal, particularly pH and coexisting ions. Uranium speciation across different pH levels was modeled with Visual MINTEQ 3.0 (Yang et al., 2024b; Figure 3d). In the pH range 3.0–7.0, positively charged uranium species (e.g., UO_2_^2^^+^, (UO_2_)_2_${\left(\text{OH}\right)}_{2}^{2+}$, (UO_2_)_3_${\left(\text{OH}\right)}_{5}^{+}$ and (UO_2_)_4_${\left(\text{OH}\right)}_{7}^{+}$) is the main species, and these species readily adsorb to negatively charged electrode surfaces. However, negatively charged uranium species (e.g., (UO_2_)_3_(OH)7^−^) gradually increased in the pH 7.0–10.0 range. As shown in Supplementary Figure S8, *S. putrefaciens* had a high removal rate of uranium in the pH 5–7 range. The growth vigor of the bacteria may be inhibited at strong acids or bases, resulting in a lower removal efficiency for uranium (Song et al., 2024). As shown in Figure 3e, *S. putrefaciens* showed remarkable resilience against interference from various ions, encompassing monovalent (K^+^, Cs^+^), divalent (Sr^2^^+^, Zn^2^^+^, Ca^2^^+^), trivalent cations (Fe3^+^, Al3^+^), as well as anions (Cl^−^, $\text{HC}{\text{O}}_{3}^{-}$).

To evaluate the uranium removal efficacy of the *S. putrefaciens*, its performance was compared with other uranium-tolerant bacteria isolated from the same uranium-bearing soil, including *Staphylococcus warneri* (*S. warneri*), Bacillus cereus (*Bacillus*), and *Alcaligenes faecalis* (*A. faecalis*). As shown in Figure 3f, the uranium removal capacities of the four indigenous strains were 93%, 52%, 63%, and 48%, respectively, demonstrating that *S. putrefaciens* was the most effective. Furthermore, the indigenous *S. putrefaciens* isolated demonstrated superior uranium removal performance compared to previously reported biosorbents (Table 1). These results indicate that *S. putrefaciens* exhibited significant uranium removal capacity, indicating that it is a highly promising microbial strain for decontaminating uranium-contaminated areas.

**Table 1:** Comparison of the indigenous *S. putrefaciens* and other biomass materials for uranium removal performance.

**Materials**	**C_0_ (mg/L)**	**Reaction time**	***Q* (mg/g)**	**References**
*S. putrefaciens*	100	120 min	259	This work
EPS	200	24 h	237	Nie et al., 2022
CSB-AO	80	240 min	100.31	He et al., 2023
*S. putrefaciens*	100	24 h	244.3	Huang et al., 2017
HMDB	10	2 min	255.5	Liao et al., 2022
*B. thuringiensis*	60	48 h	1.75	Chen et al., 2024
HAP-ZVI	100	150 min	155.8	Zeng et al., 2019
WBC	10	180 min	135.8	Lingamdinne et al., 2022
FH/Fe_3_O_4_	60	240 min	219.71	Zhu et al., 2019
MnFe_2_O_4_-biochar	30	120 min	27.61	Ahmed et al., 2021
Fe_3_O_4_-biochar	10	120 min	71.4	Philippou et al., 2019

### 3.4 Genome analysis of *S. putrefaciens*

**Figure 4:**
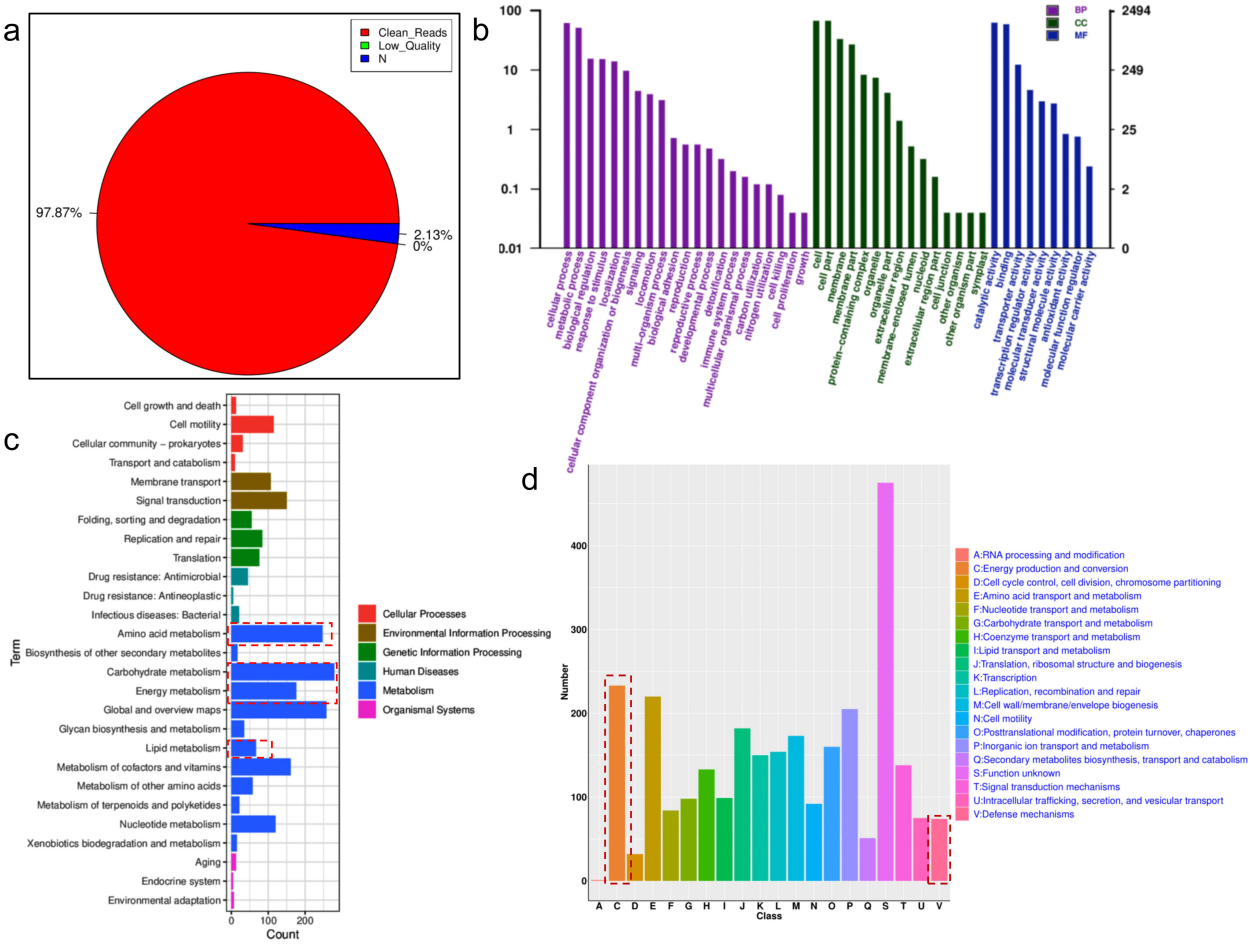
Genome analysis of *S. putrefaciens*. **(a)** Quality control analysis of raw genome-wide data; **(b)** GO annotation analysis; **(c)** KEGG annotation analysis; **(d)** COG annotation analysis.

To further reveal the function of *S. putrefaciens* at the gene level, whole genome sequencing analysis was performed. After passing the quality control, the quality control of whole genome sequencing results of *S. putrefaciens* reached 97.87% (Figure 4a), which ensured the accuracy and reliability of the subsequent data analysis. Gene prediction of *S. putrefaciens* was performed using Prodigal-2.6.2 software (https://github.com/hyattpd/prodigal/wiki) and the whole genome was characterized as shown in Supplementary Table S1. The length of genome was 4.59 Mbp with 44.37% GC content and 4005 protein coding genes containing 7 rRNAs and 97 tRNAs. Figure 4b illustrates the results of a genome-wide Gene Ontology (GO) functional annotation analysis of *S. putrefaciens*, including three main categories, biological process (BP), cellular component (CC), and molecular function (MF). In BP classification, significant enrichment of genes was observed in several functional categories related to metabolic activities, which include key biochemical processes such as substance metabolism and energy metabolism. In CC classification, their roles in cellular structures and cellular processes were revealed, such as their involvement in key cellular structures in cell membranes and organelles. Meanwhile, their importance in biological functions such as catalytic activity and molecular transporter activity was revealed in MF. In addition, the functions of these genes were further analyzed using the KEGG pathway. KEGG is a systematic analysis of gene function, genomic information database, which helps researchers to study gene and expression information as a whole network (Kanehisa and Goto, 2000). As shown in Figure 4c, *S. putrefaciens* has 2,192 genes annotated to various pathways in the KEGG database, which are mainly distributed in the areas of carbohydrate metabolism, amino acid metabolism, energy metabolism and lipid metabolism. Meanwhile, these pathways can be categorized into metabolism, environmental information processing, membrane transport and signal transduction, etc. Furthermore, the COG database was further used to analyze the direct homology classification of gene products, and the annotation results were shown in Figure 4d. In exception of 475 unknown functional genes, energy metabolism and transformation-related genes received the most annotations amounting to 233, and the defense mechanism annotations amounted to 74. In summary, the whole-genome analysis of the isolated *S. putrefaciens* strain revealed that its annotated genes play key roles in diverse biological processes. In addition, this strain exhibited remarkable genetic potential in energy metabolic conversion and resistance to external environmental stresses associated with metal reducing bacteria (Fredrickson et al., 2008; Zarei et al., 2025), supporting its physiological adaptability under uranium stress. Notably, the enrichment of energy metabolism and stress resistance genes observed in this strain aligns with previously reported adaptive traits in metal-reducing *Shewanella* species (Fang et al., 2022; Wang et al., 2023).

### 3.5 Mechanistic analysis of uranium removal from *S. putrefaciens*

**Figure 5:**
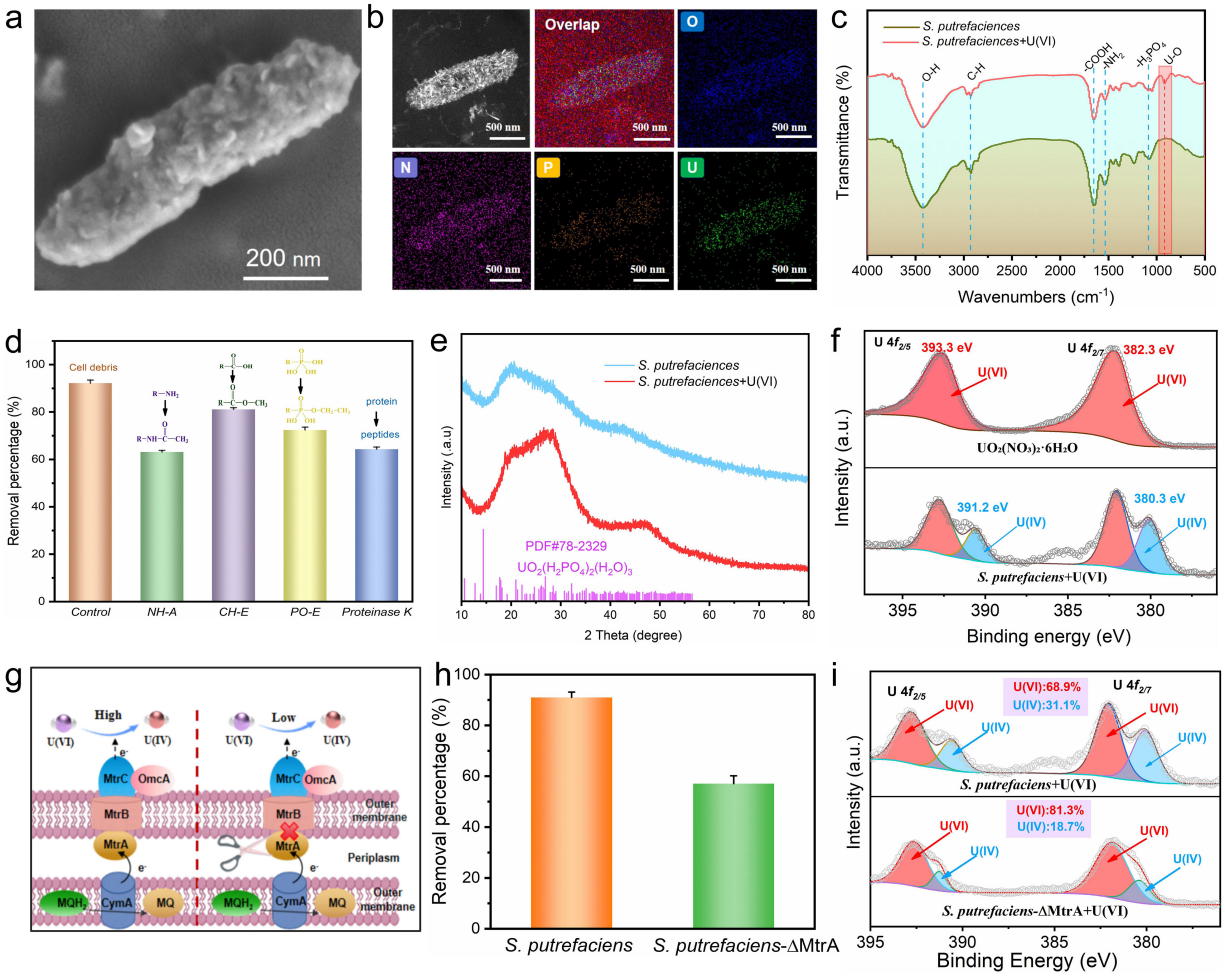
Characterization of uranium removal by *S. putrefaciens*. **(a)** SEM after uranium removal by *S. putrefaciens*; **(b)** TEM and the corresponding EDS elemental mapping images of *S. putrefaciens* with uranium; **(c)** FT-IR spectra of *S. putrefaciens* before and after uranium removal; **(d)** effect of functional groups of *S. putrefaciens* on uranium removal; **(e)** XRD spectra of *S. putrefaciens* before and after uranium removal; **(f)** XPS spectra of the U 4f of *S. putrefaciens* after uranium removal; **(g)** schematic diagram of electron transfer in *S. putrefaciens* and MtrA deficient strain; **(h)** uranium removal efficiency of *S. putrefaciens* and MtrA deficient strain; **(i)** XPS spectra of the U*4f* region from uranium loaded *S. putrefaciens* and MtrA deficient strain.

In order to determine the form of uranium presence on the *S. putrefaciens* surface and to further reveal the reaction mechanism, we analyzed the products loaded with uranium. As presented in Figure 5a, Supplementary Figure S9, the surface of the uranium-loaded bacteria showed remarkable granular deposits compared to the smooth morphology of the bacterial surface before the reaction occurred, which can be attributed to the products of uranium deposited on the surface of the cell wall. Since the cell wall of bacteria is mainly composed of peptidoglycan, lipids and proteins, these components can provide abundant organic functional groups to generate complexes with uranium during uranium enrichment. In addition, TEM and corresponding EDS elemental mapping analysis of *S. putrefaciens* after loaded with uranium revealed a uniform distribution of O, N, P, and U throughout the cell structure, with uranium accounting for 0.32% of the relative atomic content (Figure 5b, Supplementary Figure S10), confirming significant uranium enrichment. To further localize the accumulated uranium at the subcellular level, ultrathin-section TEM was performed (Supplementary Figure S11). The results clearly reveal black nanoflake-like uranium deposits distributed not only on the bacterial surface but also within the cells. This confirms that uranium is not only adsorbed onto the bacterial surface but can also be taken up internally, thereby demonstrating that the bioaccumulation process occurs concurrently with surface adsorption. Furthermore, Figure 5c displays the FT-IR spectra of both pristine *S. putrefaciens* and uranium-loaded *S. putrefaciens*. Notably, a new absorption band emerges at 917 cm^−1^ following uranium adsorption, which is assigned to the U–O vibration, confirming the formation of uranium-containing precipitates on the bacterial surface. Moreover, slight shifts were observed in the characteristic peaks of key functional groups, including –OH (3,426 cm^−1^), C–H (2,920 cm^−1^), –COOH (1,658 cm^−1^), –NH_2_ (1,525 cm^−1^), and –H_3_PO_4_ (1,081 cm^−1^), implying their participation in the uranium binding process (Yang et al., 2024a).

To further evaluate the effect of different functional groups on the removal of uranium by *S. putrefaciens*, ammonia acetylation (NH-A), carboxyl esterification (CH-E), phosphoric esterification (PO-E) and proteinase K treatments were used to shield amino groups, carboxyl groups, phosphoric groups and proteins, respectively (Zhang et al., 2018). As shown in Figure 5d, the removal efficiency of uranium decreased by 29%, 11%, 20% and 28% when the amino, carboxyl, phosphate groups and proteins were shielded compared to the control group, suggesting that these functional groups on the surface of the cell play an important role in the removal of uranium, especially the surface amino groups and the membrane proteins. Moreover, the crystal structure changes of *S. putrefaciens* after uranium removal were characterized by XRD (Figure 5e). Both before and after uranium removal, the XRD patterns of *S. putrefaciens* exhibited broad, amorphous peaks, indicating the non-crystalline nature of the biomass (Simşek et al., 2022). Following uranium loading, these peaks shifted notably yet remained featureless when compared to crystalline uranium mineral references such as UO_2_(H_2_PO_4_)_2_ (H_2_O)_3_ (PDF#78-2329). This suggests that uranium binding occurs primarily through chemical adsorption rather than crystalline precipitation, resulting in amorphous uranium complexes on the bacterial surface. To further reveal the assigned state of uranium after enrichment, U 4f XPS fitting analysis was performed on the reacted samples. As shown in Figure 5f, compared to the U(VI) peaks of UO_2_(NO_3_)_2_·6H_2_O, four characteristic peaks appeared after *S. putrefaciens* + U(VI), located at 393.3 eV, 391.2 eV, 382.3 eV and 380.3 eV, respectively, where 393.3 and 382.3 eV correspond to the binding energy of U(VI) and 391.2 eV and 380.3 eV correspond to the binding energy of U(IV) (Chen et al., 2022; Tu et al., 2023). This further confirmed that uranium captured by *S. putrefaciens* undergone an electron reduction process. Notably, *Shewanella* genus, as an electroactive microorganism, is capable of transferring electrons to the captured uranium species, with the Mtr pathway representing the most classical electron transport route (Yang et al., 2024a). Electrons of *Shewanella* are transferred through cytochrome c proteins (such as MtrA and MtrB) in the cell membrane to the extracellular space for uranium reduction, in which MtrA serves as a crucial membrane protein in this electron transfer process (Figure 5g). Therefore, to investigate the impact of transmembrane electron transport on uranium reduction mineralisation, MtrA was knocked out using homologous recombination (Supplementary Figure S12). As shown in Figure 5h, uranium removal efficiency decreased by approximately 34% in the MtrA-deficient strain compared to the wild-type. In addition, XPS spectra of U4f from uranium-loaded samples revealed a 13.1% reduction in the U(VI) peak area ratio in the MtrA-deficient strain, further confirming the critical role of the Mtr pathway in extracellular electron-mediated U(VI) reduction (Figure 5i). This pathway, which involves a suite of outer-membrane cytochromes (e.g., MtrA, MtrB, MtrC), is highly conserved among electroactive *Shewanella* species and is responsible for transferring electrons from the inner-membrane quinone pool to extracellular acceptors, including U(VI). This result is consistent with the established function of MtrA in transmembrane electron transfer in the model organism *S. oneidensis* MR-1 (Liu et al., 2021; Shi et al., 2012), confirming the conserved nature of this electron conduit in *Shewanella putrefaciens* strain. In summary, the locally isolated *S. putrefaciens* with MtrA knockout exhibited a 34% reduction in uranium removal, confirming that the Mtr-mediated extracellular electron transport pathway contributes significantly but non-exclusively. Based on our experimental evidence, we propose a synergistic multi-mechanism model: (i) biosorption via surface functional groups (e.g., carboxyl, phosphate, and amino groups); (ii) biomineralization, potentially forming amorphous uranium-phosphate complexes; (iii) potential intracellular bioaccumulation or non-MtrA-dependent reduction. Within this framework, the Mtr pathway provides a dedicated route for extracellular reduction, while biosorption and biomineralization serve as constitutive auxiliary defense mechanisms, collectively conferring the strain's resilience in uranium-rich environments.

**Figure 6:**
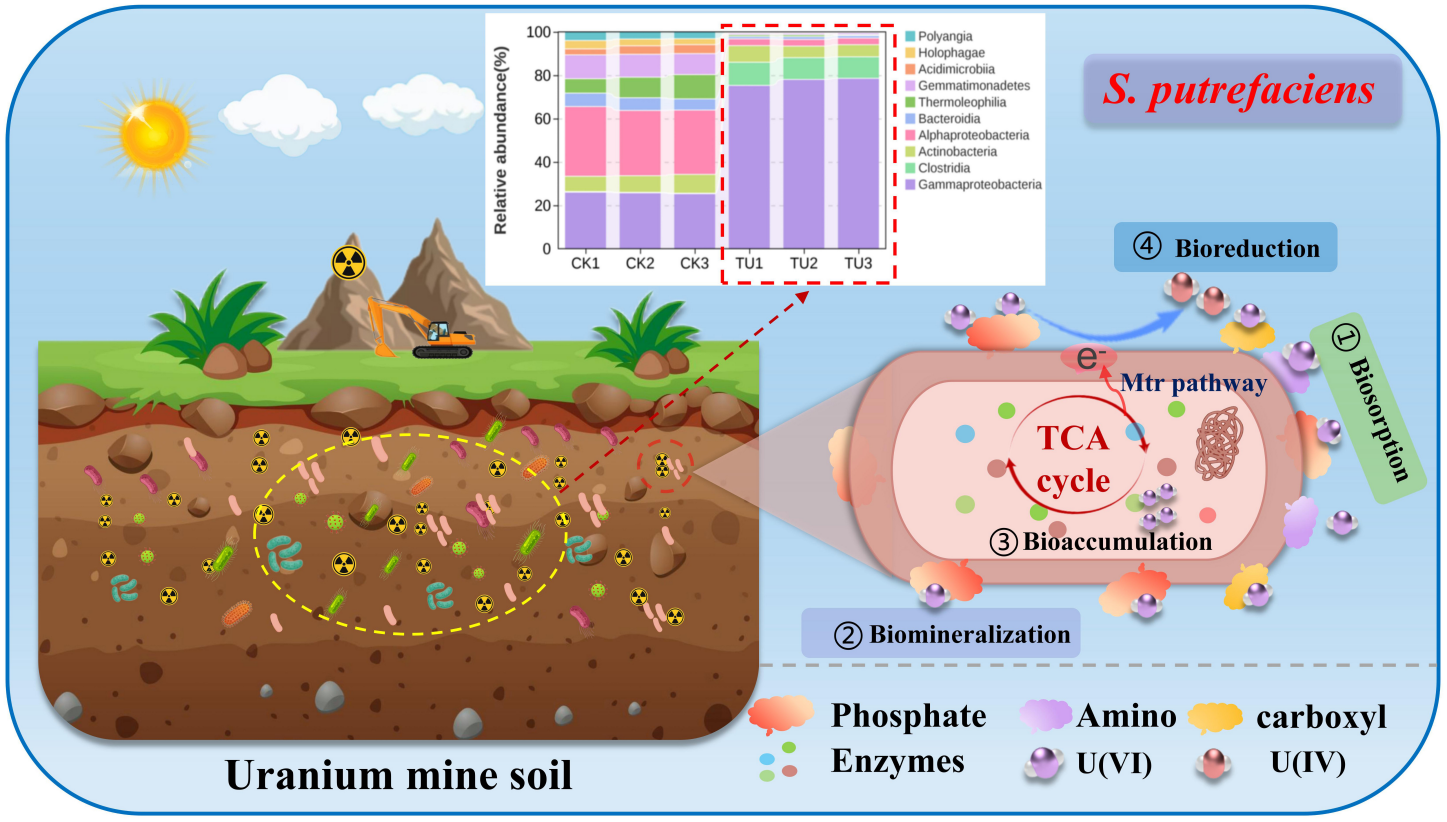
Schematic of uranium removal by *S. putrefaciens*.

Consequently, a potential mechanism for uranium removal by *S. putrefaciens* is proposed (Figure 6). The surface of *S. putrefaciens* isolated from uranium mine soil is rich in a variety of functional groups. These functional groups may act as binding sites for uranium, where amino, carboxyl and phosphate groups are able to bind to uranium by biosorption. Additionally, the absorption peak at 1,080 cm^−1^ in the FT-IR spectrum (Figure 5c), attributed to phosphate groups, and the extensive distribution of phosphorus elements in TEM-EDS (Figure 5b) both indicate that phosphate groups on the cell surface may participate in uranium coordination. Although the XRD pattern indicates an amorphous structure (Figure 5e), precluding confirmation of crystalline uranium phosphate minerals, the coexistence of U(IV)/U(VI) species observed in EDS, FT-IR, and XPS data suggests the formation of an amorphous surface complex between uranium and phosphate groups. This finding is consistent with previous reports (Lu et al., 2025; Xuan et al., 2024). At the same time, some of the uranium pass through the cell membrane into the cell interior and thus bioaccumulate. Moreover, uranium may be reduced from U(VI) to U(IV) via extracellular electrons from *S. putrefaciens*. The above four mechanisms may act synergistically to confer *S. putrefaciens*, with superior uranium removal capabilities.

## 4 Conclusion

In summary, this study employed 16S rRNA high-throughput sequencing technology to analyze the diversity and structural differences in soil microbial communities between uranium mining areas and non-mining areas. A dominant indigenous strain identified as *S. putrefaciens* was successfully isolated from uranium tailings soil, exhibiting remarkable uranium tolerance and enrichment capacity. Whole-genome sequencing and functional annotation of *S. putrefaciens* revealed significant enrichment of genes associated with energy metabolism and defense mechanisms, highlighting its genomic adaptation to radioactive environments. The uranium removal mechanisms indicates that its surface functional groups (amino, carboxyl, and phosphate groups) play a crucial role in uranium accumulation. *S. putrefaciens* achieves efficient uranium removal through multiple pathways including biosorption, bioreduction, bioaccumulation, and biomineralization. This study not only elucidates the uranium accumulation mechanism of *S. putrefaciens* but also provides important references for analyzing microbial diversity in uranium mining areas and for *in situ* ecological remediation of uranium pollution based on indigenous microorganisms.

## Data Availability

The 16S rRNA gene sequence of Shewanella putrefaciens strain A-1 presented in this study has been deposited in the NCBI GenBank database under accession ID PX705773.

## References

[B1] Ahmed W. Mehmood S. Núñez-Delgado A. Ali S. Qaswar M. Khan Z. H. . (2021). Utilization of *Citrullus lanatus* L. seeds to synthesize a novel MnFe_2_O_4_-biochar adsorbent for the removal of U(VI) from wastewater: insights and comparison between modified and raw biochar. Sci. Total Environ. 771:144955. doi: 10.1016/j.scitotenv.2021.144955 33736137

[B2] Bach E. M. Williams R. J. Hargreaves S. K. Yang F. Hofmockel K. S. (2018). Greatest soil microbial diversity found in micro-habitats. Soil Biol. Biochem. 118, 217–226. doi: 10.1016/j.soilbio.2017.12.018

[B3] Banala U. K. Das N. P. I. Toleti S. R. (2021). Microbial interactions with uranium: towards an effective bioremediation approach. Environ. Technol. Innov. 21:101254. doi: 10.1016/j.eti.2020.101254

[B4] Bhatti T. M. Mateen A. Amin M. Malik K. A. Khalid A. M. (1991). Spectrophotometric determination of uranium(VI) in bacterial leach liquors using arsenazo-III. J. Chem. Technol. Biotechnol. 52, 331–341. doi: 10.1002/jctb.280520306

[B5] Bondici V. F. Lawrence J. R. Khan N. H. Hill J. E. Yergeau E. Wolfaardt G. M. . (2013). Microbial communities in low permeability, high pH uranium mine tailings: characterization and potential effects. J. Appl. Microbiol. 114, 1671–1686. doi: 10.1111/jam.12180 23448257

[B6] Chen H. Sheng Y. Wang S. Chen Y. Qiao Z. Guo H. . (2025). Uranium contamination mediating soil and ore microbial community assembly at four mining sites, South China. Front. Microbiol. 16:1553072. doi: 10.3389/fmicb.2025.1553072 40046305 PMC11879985

[B7] Chen L. Liu J. Zhang W. Zhou J. Luo D. Li Z. (2021). Uranium (U) source, speciation, uptake, toxicity and bioremediation strategies in soil-plant system: a review. J. Hazard. Mater. 413:125319. doi: 10.1016/j.jhazmat.2021.125319 33582470

[B8] Chen S. Gong J. Cheng Y. Guo Y. Li F. Lan T. . (2024). The biochemical behavior and mechanism of uranium (VI) bioreduction induced by natural *Bacillus thuringiensis*. J. Environ. Sci. 136, 372–381. doi: 10.1016/j.jes.2022.12.001 37923447

[B9] Chen T. Liu T. Pang B. Ding T. Zhang W. Shen X. . (2022). Actinide-uranium single-atom catalysis for electrochemical nitrogen fixation. Sci. Bull. 67, 2001–2012. doi: 10.1016/j.scib.2022.09.001 36546210

[B10] Dhal P. K. Islam E. Kazy S. K. Sar P. (2011). Culture-independent molecular analysis of bacterial diversity in uranium-ore/-mine waste-contaminated and non-contaminated sites from uranium mines. 3 Biotech 1, 261–272. doi: 10.1007/s13205-011-0034-4 22558545 PMC3339583

[B11] Domeignoz-Horta L. A. Pold G. Erb H. Sebag D. Verrecchia E. Northen T. . (2023). Substrate availability and not thermal acclimation controls microbial temperature sensitivity response to long-term warming. Global Change Biol. 29, 1574–1590. doi: 10.1111/gcb.16544 36448874

[B12] Fang L. Li Y. Li Y. Cao Y. Song H. (2022). Transcriptome analysis to identify crucial genes for reinforcing flavins-mediated extracellular electron transfer in *Shewanella oneidensis*. Front. Microbiol. 13:852527. doi: 10.3389/fmicb.2022.852527 35722328 PMC9198578

[B13] Fredrickson J. K. Romine M. F. Beliaev A. S. Auchtung J. M. Driscoll M. E. Gardner T. S. . (2008). Towards environmental systems biology of Shewanella. Nat. Rev. Microbiol. 6, 592–603. doi: 10.1038/nrmicro1947 18604222

[B14] Guo Y. Li X. Tu H. Zeng Q. Chen S. Gong J. . (2025). Insight into the complexation of uranium with Bacillus sp. dwc-2 by multi-spectroscopic approaches: FT-IR, TRLF and XAFS spectroscopies. J. Mol. Struct. 1321:140145. doi: 10.1016/j.molstruc.2024.140145

[B15] Handelsman J. Wackett L. P. (2002). Ecology and industrial microbiology: microbial diversity—sustaining the Earth and industry. Curr. Opin. Microbiol. 5, 237–239. doi: 10.1016/S1369-5274(02)00331-4 12057675

[B16] Hau H. H. Gralnick J. A. (2007). Ecology and biotechnology of the genus Shewanella. Annu. Rev. Microbiol. 61, 237–258. doi: 10.1146/annurev.micro.61.080706.093257 18035608

[B17] He Y. Wang Y. Cai C. Yang G. Zhou L. Ran G. . (2023). Cotton stalk derived carbon pretreated by microbial fermentation for selective uranium extraction. J. Radioanal. Nucl. Chem. 332, 2149–2158. doi: 10.1007/s10967-023-08827-2

[B18] Huang W. Nie X. Dong F. Ding C. Huang R. Qin Y. . (2017). Kinetics and pH-dependent uranium bioprecipitation by Shewanella putrefaciens under aerobic conditions. J. Radioanal. Nucl. Chem. 312, 531–541. doi: 10.1007/s10967-017-5261-7

[B19] Jiang R. Wang M. Chen W. Li X. Balseiro-Romero M. (2020). Changes in the integrated functional stability of microbial community under chemical stresses and the impacting factors in field soils. Ecol. Indic. 110:105919. doi: 10.1016/j.ecolind.2019.105919

[B20] Kanehisa M. Goto S. (2000). KEGG: Kyoto encyclopedia of genes and genomes. Nucleic Acids Res. 28, 27–30. doi: 10.1093/nar/28.1.27 10592173 PMC102409

[B21] Kolmogorov M. Yuan J. Lin Y. Pevzner P. A. (2019). Assembly of long, error-prone reads using repeat graphs. Nat. Biotechnol. 37, 540–546. doi: 10.1038/s41587-019-0072-8 30936562

[B22] Lashani E. Amoozegar M. A. Turner R. J. Moghimi H. (2023). Use of microbial consortia in bioremediation of metalloid polluted environments. Microorganisms 11:891. doi: 10.3390/microorganisms11040891 37110315 PMC10143001

[B23] Li N. Wang Y. Zhou L. Fu D. Chen T. Chen X. . (2024). The joint action of biochar and plant roots on U-stressed soil remediation: insights from bacteriomics and metabolomics. J. Hazard. Mater. 461:132635. doi: 10.1016/j.jhazmat.2023.132635 37793252

[B24] Li Q. Xiong Z. Xiang P. Zhou L. Zhang T. Wu Q. . (2024). Effects of uranium mining on soil bacterial communities and functions in the Qinghai-Tibet plateau. Chemosphere 347:140715. doi: 10.1016/j.chemosphere.2023.140715 37979803

[B25] Li R. Zhang L. Chen Y. Xia Q. Liu D. Huang Y. . (2024). Oxidation of biogenic U(IV) in the presence of bioreduced clay minerals and organic ligands. Environ. Sci. Technol. 58, 1541–1550. doi: 10.1021/acs.est.3c07385 38199960

[B26] Li X. Ding C. Liao J. Du L. Sun Q. Yang J. . (2017). Microbial reduction of uranium (VI) by Bacillus sp. dwc-2: a macroscopic and spectroscopic study. J. Environ. Sci. 53, 9–15. doi: 10.1016/j.jes.2016.01.030 28372765

[B27] Liao J. He X. Zhang Y. Zhu W. Zhang L. He Z. (2022). Bismuth impregnated biochar for efficient uranium removal from solution: adsorption behavior and interfacial mechanism. Sci. Total Environ. 819:153145. doi: 10.1016/j.scitotenv.2022.153145 35038520

[B28] Lin T. Chen T. Jiao C. Zhang H. Hou K. Jin H. . (2024). Ion pair sites for efficient electrochemical extraction of uranium in real nuclear wastewater. Nat. Commun. 15:4149. doi: 10.1038/s41467-024-48564-y 38755163 PMC11099191

[B29] Lingamdinne L. P. Choi J. -S. Angaru G. K. R. Karri R. R. Yang J. -K. Chang Y. -Y. . (2022). Magnetic-watermelon rinds biochar for uranium-contaminated water treatment using an electromagnetic semi-batch column with removal mechanistic investigations. Chemosphere 286:131776. doi: 10.1016/j.chemosphere.2021.131776 34371355

[B30] Liu D. -F. Huang X. -N. Cheng R. -F. Min D. Cheng L. Zhao F. . (2021). Anaerobic respiration on nitarsone in aquatic environments by *Shewanella oneidensis* MR-1 lacking known C·as lyases. ACS ESandT Water 1, 603–612. doi: 10.1021/acsestwater.0c00124

[B31] Liu Y. Zhao B. He P. Wang Z. Tang K. Mou Z. . (2024). Cinnamic acid: a low-toxicity natural bidentate ligand for uranium decorporation. Inorg. Chem. 63, 7464–7472. doi: 10.1021/acs.inorgchem.4c00610 38598182

[B32] Lu X. Zhang Y. -Y. Cheng W. Liu Y. Li Q. Li X. . (2024). Chelating effect of siderophore desferrioxamine-B on uranyl biomineralization mediated by *Shewanella putrefaciens*. Environ. Sci. Technol. 58, 3974–3984. doi: 10.1021/acs.est.3c05753 38306233

[B33] Lu X. Zhang Y. -Y. Cheng W. Liu Y. Li Q. Li X. . (2025). Correction to “chelating effect of siderophore desferrioxamine-B on uranyl biomineralization mediated by *Shewanella putrefaciens*. Environ. Sci. Technol. 59, 9360–9361. doi: 10.1021/acs.est.5c04349 40314384

[B34] Lv Y. Cheng R. Yang G. Liu W. Zhang J. Yu X. . (2023). Self-oxidation cycle of oxidized red phosphorus for regeneration of binding sites enables efficient uranium extraction in tributylphosphate-kerosene system. Chem. Eng. J. 460:141834. doi: 10.1016/j.cej.2023.141834

[B35] Müller S. von Bonin S. Schneider R. Krüger M. Quick S. Schröttner P. (2022). *Shewanella putrefaciens*, a rare human pathogen: a review from a clinical perspective. Front Cell Infect. Microbiol. 12:1033639. doi: 10.3389/fcimb.2022.1033639 36817694 PMC9933709

[B36] Nearing J. T. Douglas G. M. Hayes M. G. MacDonald J. Desai D. K. Allward N. . (2022). Microbiome differential abundance methods produce different results across 38 datasets. Nat. Commun. 13:342. doi: 10.1038/s41467-022-28034-z 35039521 PMC8763921

[B37] Nie X. Lin Q. Dong F. Cheng W. Ding C. Wang J. . (2022). Surface biomineralization of uranium onto Shewanella putrefaciens with or without extracellular polymeric substances. Ecotoxicol. Environ. Saf. 241:113719. doi: 10.1016/j.ecoenv.2022.113719 35691198

[B38] Okamoto A. Hashimoto K. Nealson K. H. Nakamura R. (2013). Rate enhancement of bacterial extracellular electron transport involves bound flavin semiquinones. Proc. Natl. Acad. Sci. 110, 7856–7861. doi: 10.1073/pnas.1220823110 23576738 PMC3651484

[B39] Pajares S. Bohannan B. J. (2016). Ecology of nitrogen fixing, nitrifying, and denitrifying microorganisms in tropical forest soils. *Front*. Microbiol. 7:1045. doi: 10.3389/fmicb.2016.01045 PMC4932190 27468277

[B40] Philippou K. Anastopoulos I. Dosche C. Pashalidis I. (2019). Synthesis and characterization of a novel Fe_3_O_4_-loaded oxidized biochar from pine needles and its application for uranium removal. Kinetic, thermodynamic, and mechanistic analysis. J. Environ. Manage. 252:109677. doi: 10.1016/j.jenvman.2019.109677 31629175

[B41] Qu M. Guang X. Liu H. Zhao Y. Huang B. (2022). Additional sampling using *in-situ* portable X-ray fluorescence (PXRF) for rapid and high-precision investigation of soil heavy metals at a regional scale. Environ. Pollut. 292:118324. doi: 10.1016/j.envpol.2021.118324 34637827

[B42] Richardson D. B. Rage E. Demers P. A. Do M. T. Fenske N. Deffner V. . (2022). Lung cancer and radon: pooled analysis of uranium miners hired in 1960 or later. Environ. Health Perspect. 130:057010. doi: 10.1289/EHP10669 35604341 PMC9126132

[B43] Shi L. Rosso K. M. Clarke T. A. Richardson D. J. Zachara J. M. Fredrickson J. K. (2012). Molecular underpinnings of Fe(III) oxide reduction by *Shewanella oneidensis* MR-1. Front. Microbiol. 3:50. doi: 10.3389/fmicb.2012.00050 22363328 PMC3279761

[B44] Simşek S. Derin Y. Kaya S. Senol Z. M. Katin K. P. Özer A. . (2022). High-performance material for the effective removal of uranyl ion from solution: computationally supported experimental studies. Langmuir 38, 10098–10113. doi: 10.1021/acs.langmuir.2c00978 35946525 PMC9404547

[B45] Song X. Li J. Xiong Z. Sha H. Wang G. Liu Q. . (2024). Effects of detoxifying substances on uranium removal by bacteria isolated from mine soils: performance, mechanisms, and bacterial communities. Microb. Ecol. 87:111. doi: 10.1007/s00248-024-02428-6 39231820 PMC11374843

[B46] Tong S. Cao G. Zhang Z. Zhang J. Yan X. (2023). Soil microbial community diversity and distribution characteristics under three vegetation types in the Qilian Mountains, China. J. Arid. Land 15, 359–376. doi: 10.1007/s40333-023-0006-7

[B47] Tu B. Yu K. Fu D. Zhou L. Wang R. Jiang X. . (2023). Amino-rich Ag-NWs/NH2-MIL-125(Ti) hybrid heterostructure via LSPR effect for photo-assist uranium extraction from fluorine-containing uranium wastewater without sacrificial agents. Appl. Catal. B 337:122965. doi: 10.1016/j.apcatb.2023.122965

[B48] Turick C. E. Shimpalee S. Satjaritanun P. Weidner J. Greenway S. (2019). Convenient non-invasive electrochemical techniques to monitor microbial processes: current state and perspectives. Appl. Microbiol. Biotechnol. 103, 8327–8338. doi: 10.1007/s00253-019-10091-y 31478059 PMC6800409

[B49] Verma S. Kuila A. (2019). Bioremediation of heavy metals by microbial process. Environ. Technol. Innov. 14:100369. doi: 10.1016/j.eti.2019.100369

[B50] Walker B. J. Abeel T. Shea T. Priest M. Abouelliel A. Sakthikumar S. . (2014). Pilon: an integrated tool for comprehensive microbial variant detection and genome assembly improvement. PLoS ONE 9:e112963. doi: 10.1371/journal.pone.0112963 25409509 PMC4237348

[B51] Wang J. Hu H. Lin K. Wei X. Beiyuan J. Xiong X. . (2024). Pb isotopic fingerprinting of uranium pollution: new insight on uranium transport in stream-river sediments. J. Hazard. Mater. 472:134417. doi: 10.1016/j.jhazmat.2024.134417 38691992

[B52] Wang R. Li M. Liu T. Li X. Zhou L. Tang L. . (2022). Encapsulating carbon-coated nano zero-valent iron particles with biomass-derived carbon aerogel for efficient uranium extraction from uranium-containing wastewater. J. Clean. Prod. 364:132654. doi: 10.1016/j.jclepro.2022.132654

[B53] Wang X. Zhao L. Zhang X. Wei Y. Lu A. Zhou J. . (2025). Exploring functional microbiota for uranium sequestration in Zoige uranium mine soil. Microbiol. Spectrum 16:e02517–e02524. doi: 10.1128/spectrum.02517-24 40237515 PMC12131822

[B54] Wang X. -Y. Yan J. Xie J. (2023). Applications of genomics, metabolomics, Fourier transform infrared in the evaluation of spoilage targets of *Shewanella putrefaciens* from spoiled bigeye tuna. J. Agric. Food. Chem. 71, 9558–9568. doi: 10.1021/acs.jafc.3c02113 37306251

[B55] Wick R. R. Judd L. M. Gorrie C. L. Holt K. E. (2017). Unicycler: resolving bacterial genome assemblies from short and long sequencing reads. PLoS Comput. Biol. 13:e1005595. doi: 10.1371/journal.pcbi.1005595 28594827 PMC5481147

[B56] Xi L. He S. Qin Y. Chen L. Tan S. Chen S. (2024). Biosynthesis of biogenic ferrous sulfide as a potential electron shuttle for enhanced Cr(VI) removal by *Shewanella oneidensis* MR-1. J. Water Process Eng. 57:104725. doi: 10.1016/j.jwpe.2023.104725

[B57] Xiang Y. Dong Y. Zhao S. Ye F. Wang Y. Zhou M. . (2020). Microbial distribution and diversity of soil around a manganese mine area. Water Air Soil Pollut. 231:506. doi: 10.1007/s11270-020-04878-3

[B58] Xuan G. -X. Zhang G. -H. Cheng W. -C. Ma C. -Y. Li Q. -R. Liu E. -T. . (2024). Uranium speciation and distribution on the surface of *Shewanella putrefaciens* in the presence of inorganic phosphate and zero-valent iron under anaerobic conditions. *Sci*. Total Environ. 912:169438. doi: 10.1016/j.scitotenv.2023.169438 38135082

[B59] Yang C. Aslan H. Zhang P. Zhu S. Xiao Y. Chen L. . (2020). Carbon dots-fed *Shewanella oneidensis* MR-1 for bioelectricity enhancement. Nat. Commun. 11:1379. doi: 10.1038/s41467-020-14866-0 32170166 PMC7070098

[B60] Yang G. Wei L. Lv Y. He Y. Zhu B. Wu X. . (2024a). Photo-assisted enhancement of uranium mine wastewater purification by a self-assembled *Shewanella putrefaciens*-CdS biohybrid system. ACS Mater. Lett. 6, 4606–4616. doi: 10.1021/acsmaterialslett.4c00594

[B61] Yang G. Wei L. Wang X. Wu X. He Y. Li G. . (2024b). Enhancing commercially iron powder electron transport by surface biosulfuration to achieve uranium extraction from uranium ore wastewater. Inorg. Chem. 63, 1378–1387. doi: 10.1021/acs.inorgchem.3c03906 38164710

[B62] You W. Peng W. Tian Z. Zheng M. (2021). Uranium bioremediation with U (VI)-reducing bacteria. Sci. Total Environ. 798:149107. doi: 10.1016/j.scitotenv.2021.149107 34325147

[B63] Zarei M. Ghasemi R. Mir-Derikvand M. Hosseinpour H. Samani T.R. Fatemi F. (2025). The enhancement of mtrABDEF gene expressions in *Shewanella azerbaijanica*, through acclimation in high uranium concentrations. Radiochim. Acta 113, 457–470. doi: 10.1515/ract-2024-0362

[B64] Zeng H. Lu L. Gong Z. Guo Y. Mo J. Zhang W. . (2019). Nanoscale composites of hydroxyapatite coated with zero valent iron: preparation, characterization and uranium removal. J. Radioanal. Nucl. Chem. 320, 165–177. doi: 10.1007/s10967-019-06451-7

[B65] Zhang J. Song H. Chen Z. Liu S. Wei Y. Huang J. . (2018). Biomineralization mechanism of U(VI) induced by *Bacillus cereus* 12-2: the role of functional groups and enzymes. Chemosphere 206, 682–692. doi: 10.1016/j.chemosphere.2018.04.181 29783053

[B66] Zhang Y. Mei B. Tian X. Jia L. Zhu W. (2023). Remediation of uranium(VI)-containing wastewater based on a novel graphene oxide/hydroxyapatite membrane. J. Membr. Sci. 675:121543. doi: 10.1016/j.memsci.2023.121543

[B67] Zhang Z.-Y. Qiang F.-F. Liu G.-Q. Liu C.-H. Ai N. (2023). Distribution characteristics of soil microbial communities and their responses to environmental factors in the sea buckthorn forest in the water-wind erosion crisscross region. Front. Microbiol. 3:1098952. doi: 10.3389/fmicb.2022.1098952 36704571 PMC9871601

[B68] Zhou X. Chen X. Qi X. Zeng Y. Guo X. Zhuang G. . (2023). Soil bacterial communities associated with multi-nutrient cycling under long-term warming in the alpine meadow. Front. Microbiol. 4:113618711361871136187. doi: 10.3389/fmicb.2023.1136187 36910214 PMC9995882

[B69] Zhu W. Lei J. Li Y. Dai L. Chen T. Bai X. . (2019). Procedural growth of fungal hyphae/Fe_3_O_4_/graphene oxide as ordered-structure composites for water purification. Chem. Eng. J. 355, 777–783. doi: 10.1016/j.cej.2018.08.215

