# Supplementary material for: Insights into uranium enrichment of indigenous electroactive Shewanella putrefaciens
Source: Front Microbiol. 2026 Jan 29;16:1731432. doi: 10.3389/fmicb.2025.1731432 (PMC12894389; doi:10.3389/fmicb.2025.1731432)
Supplement: Supplementary file 1 [file Data_Sheet_1.docx]

**Supporting Information for**

**Insights into uranium enrichment of indigenous electroactive *Shewanella putrefaciens***

Guolin Yang^1*^, Ling Wei^2^, Liang Liu^1^, Bo Mu^1*^, Tao Chen^3*^

^1^Institute of Basic Medicine and Forensic Medicine, Nuclear Medicine and Radiation Safety Key Laboratory of Sichuan Province, North Sichuan Medical College, Nanchong, Sichuan, 637100, P. R. China

^2^Department of Agricultural Science and Technology, Nanchong Vocation and Technical College, Nanchong, Sichuan, 637131, P. R. China

^3^CAEA Innovation Center of Nuclear Environmental Safety Technology, School of National Defense & Nuclear Science and Technology, Southwest University of Science and Technology, Mianyang, Sichuan, 621010, P. R. China

^*^To whom correspondence should be addressed.

E-mail: yangguolin@nsmc.edu.cn (Guolin Yang)

**Section S1: Identification of strain 16S rDNA gene sequencing**

The isolated bacterial strain was cultured overnight in LB broth at 30°C with shaking. Genomic DNA was extracted from the pure culture using a commercial bacterial DNA extraction kit (Sangon Biotech, SK8255) according to the manufacturer’s instructions. The nearly full-length 16S rRNA gene was amplified via PCR using the universal bacterial primers 27F (5′-AGAGTTTGATCMTGGCTCAG-3′) and 1492R (5′-GGTTACCTTGTTACGACTT-3′), yielding an amplicon of approximately 1.5k bp. The 25 μL PCR reaction mixture contained: 0.5 μL template DNA, 2.5 μL 10× PCR Buffer, 1 μL of 2.5 mM dNTP mix, 0.2 μL Taq DNA polymerase, 0.5 μL of each primer (10 μM), and nuclease-free water to volume. Thermal cycling conditions were: initial denaturation at 95°C for 5 min; 30 cycles of denaturation at 95°C for 30 s, annealing at 57°C for 30 s, and extension at 72°C for 90 s; followed by a final extension at 72°C for 5 min. PCR products were verified by 1% agarose gel electrophoresis, purified using a PCR purification kit (Sangon Biotech), and then subjected to Sanger sequencing by Sangon Biotech Co., Ltd. (Shanghai). The obtained sequence was compared against the NCBI nucleotide database using the BLAST algorithm (https://blast.ncbi.nlm.nih.gov). A phylogenetic tree was constructed with the retrieved related sequences using MEGA11 software, applying the maximum-likelihood method with 1000 bootstrap replicates.

**Section S2: Characterization**

The *S. putrefaciens* was fixed overnight with 2.5% glutaraldehyde, and dehydrated progressively in 25, 50, 75, 90 and 100% ethanol. Scanning electron microscopy (SEM, Hitachi SU8020) and high-resolution transmission electron microscopy (TEM, FEI Tecnai G2 F20 S-TWIN) were used to examine the morphologies of the samples. EDS (X-MaxN, Oxford Instruments) was used to examine the surface elemental compositions. A Thermo ESCALA 250 XPS spectrometer equipment was used to perform XPS measurements utilizing Al K radiation (powered at 15 kV) in the constant analyzer energy mode. The X-ray diffraction (XRD) patterns were performed using the Philips X'Pert Pro Super diffractometer. The Raman pattern was recorded using a Renishaw System 2000 spectrometer. Fourier transform infrared spectroscopy (FT-IR) was used to determine the functional group. Electrochemical measurement was conducted by an electrochemical workstation (CH Instruments Inc, Austin, TX) with Ag/AgCl and Pt wire as the reference and counter electrode, respectively.


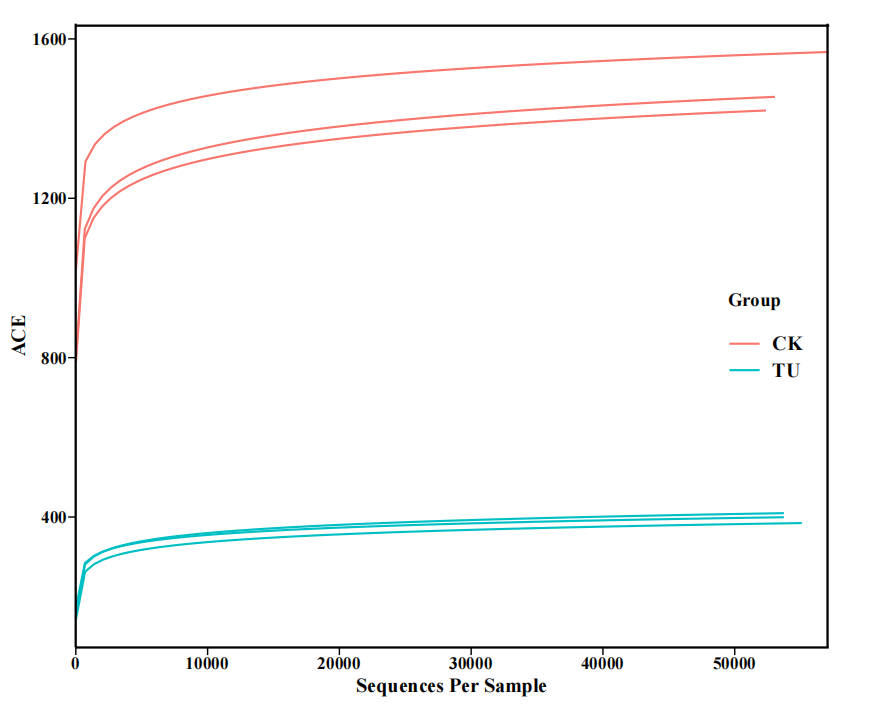


**Figure S1.** Dilution curve of soil bacterial community.


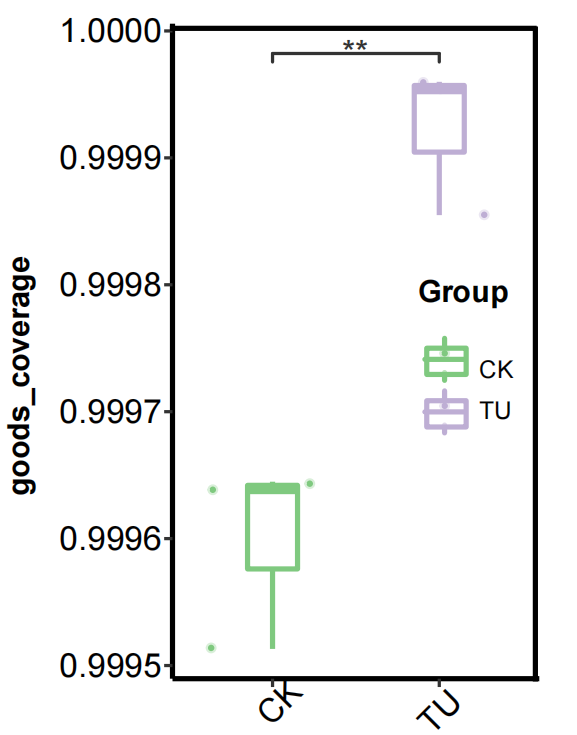


**Figure S2.** Sequencing depth.


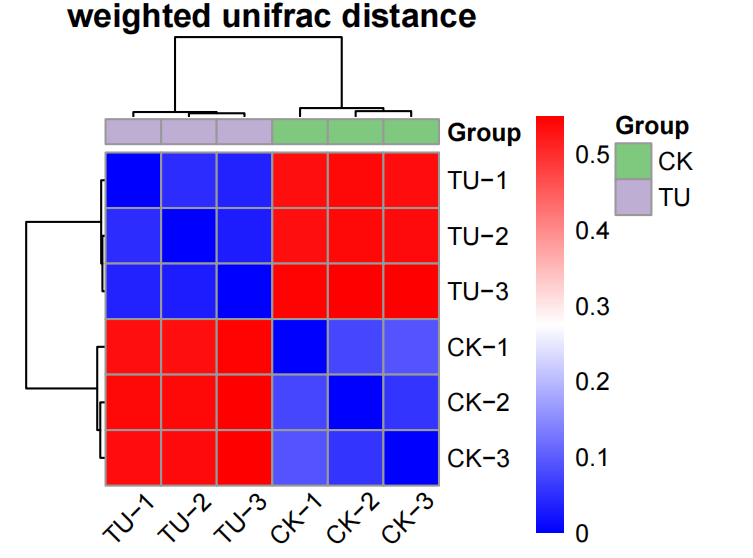


**Figure S3.** Weighted-unifrac distance analysis.


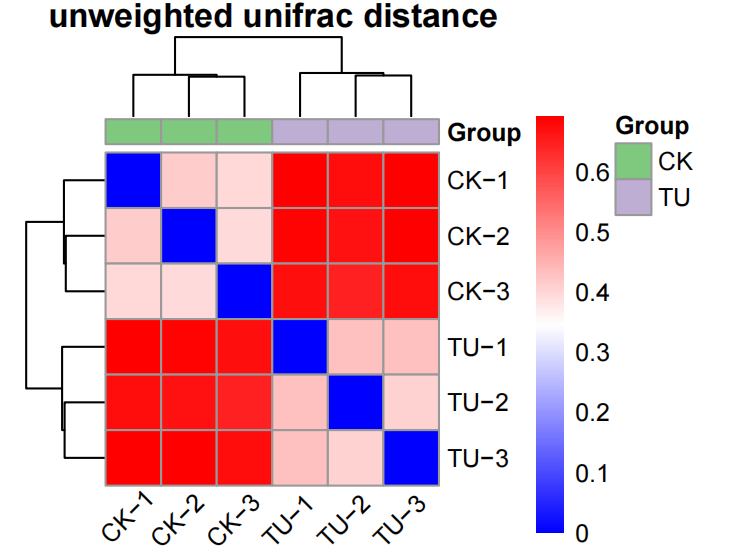


**Figure S4.** Unweighted-unifrac distance analysis.


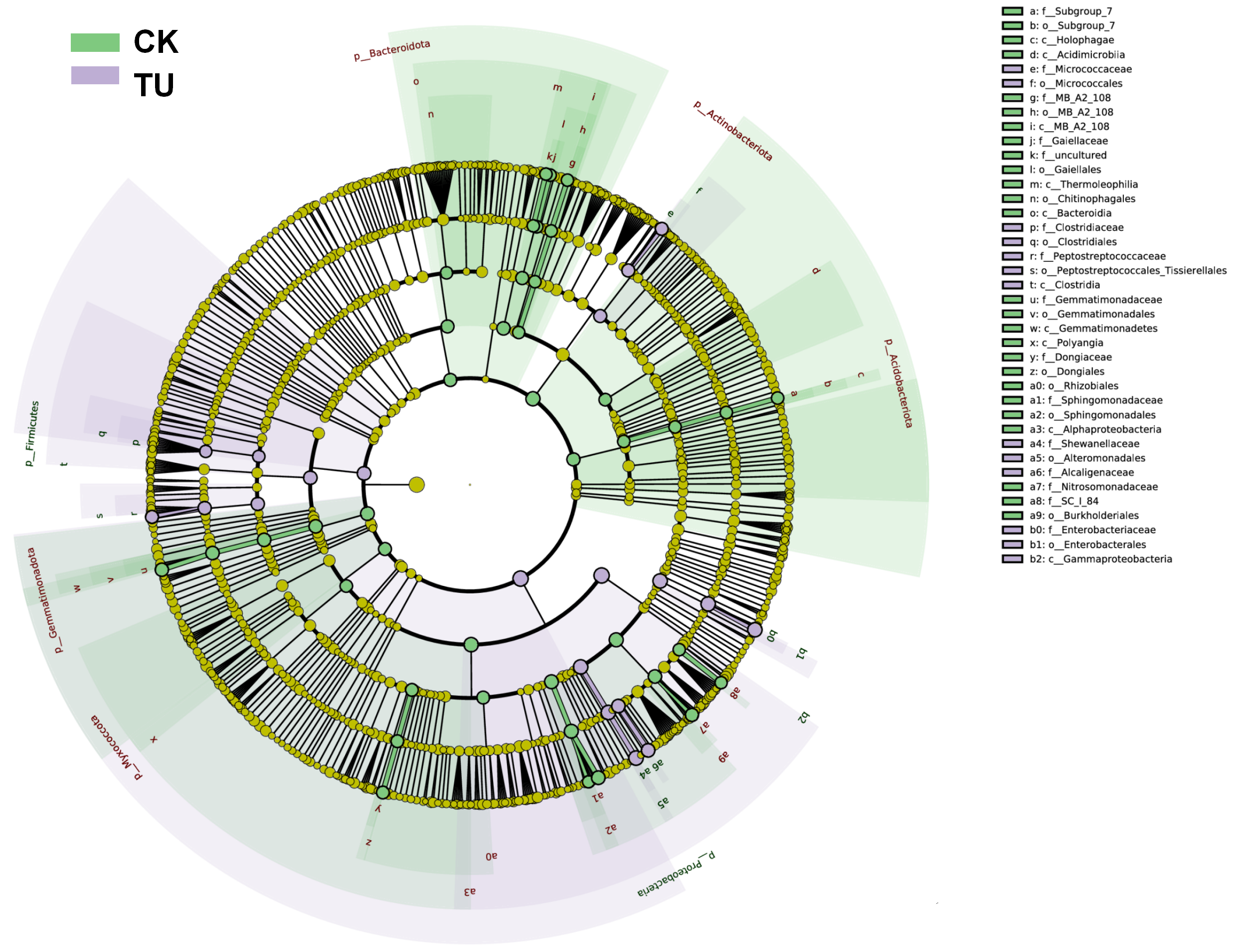


**Figure S5.** LEfSe analysis of bacterial communities.


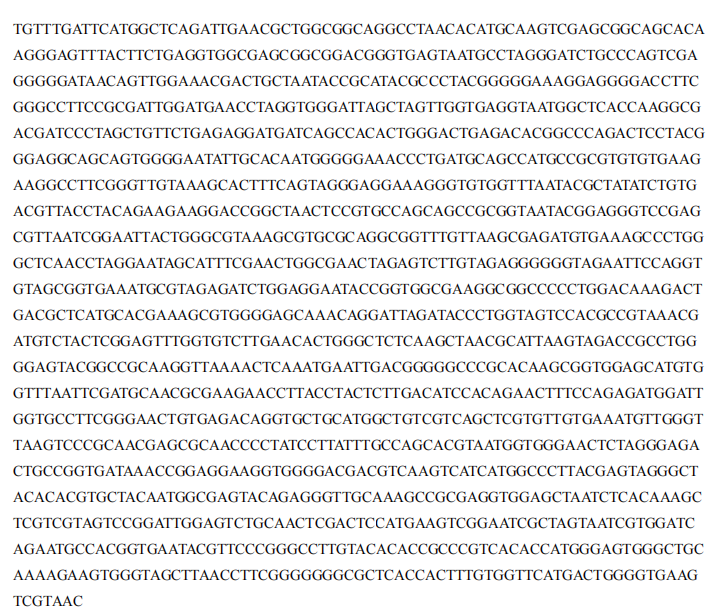


**Figure S6.** 16S rRNA gene sequence of A-1*.*





**Figure S7.** Growth curve of *S. putrefaciens.*





**Figure S8.** Effect of pH on *S. putrefaciens* removal of uranium.


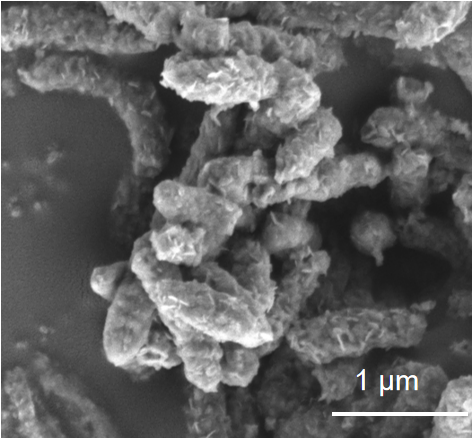


**Figure S9.** SEM after uranium removal by *S. putrefaciens*.


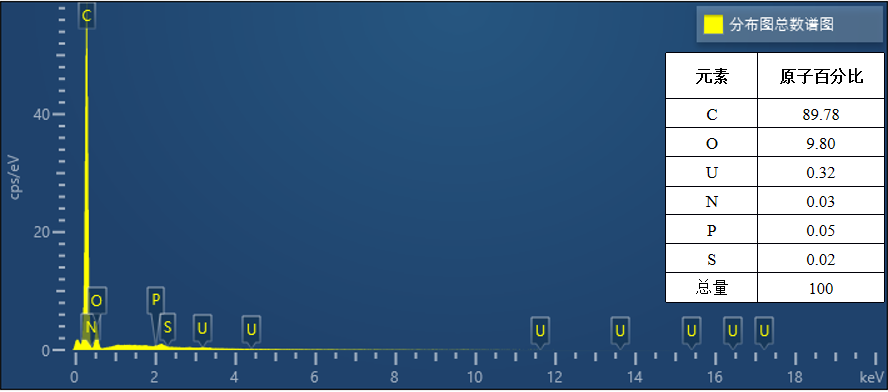


**Figure S10.** Analysis of EDS-Maping of removal uranium by *S. putrefaciens.*


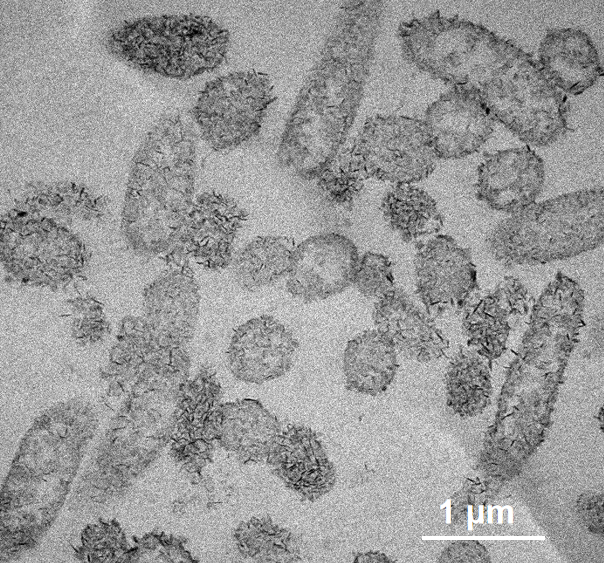


**Figure S11.** TEM after uranium removal by *S. putrefaciens.*


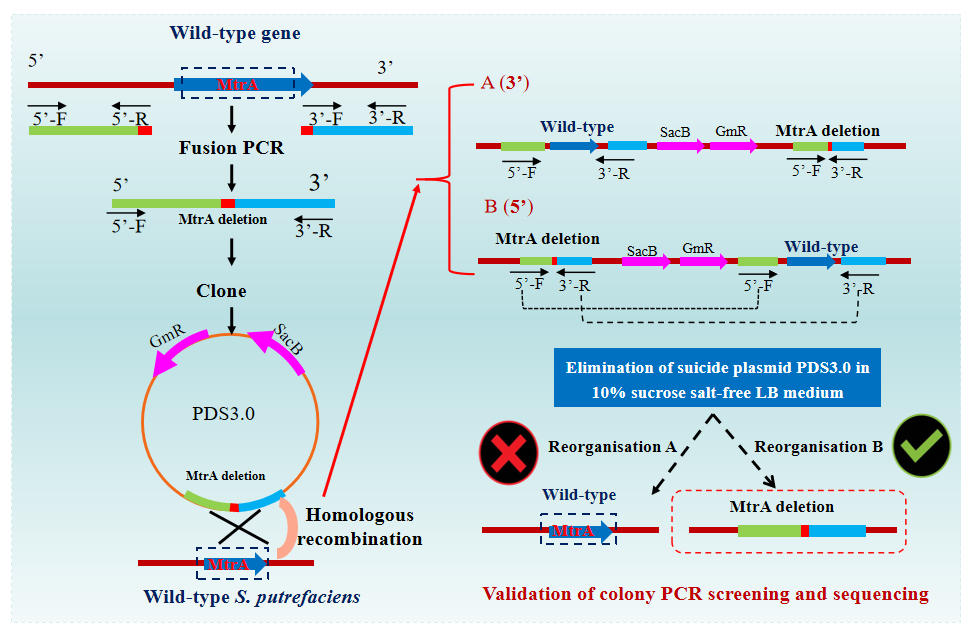


**Figure S12.** Schematic illustration of the generation of MtrA gene knockout mutants using homologous recombination in *S. putrefaciens*.

**Table S1.** General characterization of the whole genome of *S. putrefaciens.*

| **Category** | **Property** |
| --- | --- |
| Genome Size (Mbp) | 4.59 |
| GC content(%) | 44.37 |
| Protein coding genes | 4005 |
| rRNA genes | 7 |
| tRNA genes | 97 |
| Total gene length | 3973576 |
| Average gene length | 966 |
| GC contentin gene region | 45 |
| Gene density (genes/Mb) | 916 |
| Gene/Geonme (%) | 86 |
| Intergenetic region length | 617495 |
